# Evolution characteristics and obstacle factors of rural resilience in Chinese minority areas in the background of rural tourism and COVID-19

**DOI:** 10.1038/s41598-025-94186-9

**Published:** 2025-03-19

**Authors:** Jilin Wu, Derong Guo, Jinyou Zuo, Jing Yang, Shuiliang Liu

**Affiliations:** 1 School of Civil Engineering and Architecture, Jishou University, Zhangjiajie, 427000 Hunan China; 2 Rural Planning and Development Research Center of Wuling Mountain Area, Zhangjiajie, 427000 Hunan China; 3 School of Tourism, Jishou University, Zhangjiajie, 427000 Hunan China; 4 School of Arts and Communication, China University of Geosciences, Wuhan, 430074 Hubei China

**Keywords:** Tourism village resilience, Production-living-ecology, Livelihood type, Evolutionary characteristics, Obstacle factors, Xiangxi Prefecture, Sustainability, Environmental economics

## Abstract

Rural tourism and COVID-19 have brought significant impacts and lasting challenges to the resilience development of rural territorial systems. To improve the livelihoods of farmers and promote the sustainable development of tourism village systems, it is of great significance to clarify the characteristics of, and obstacle factors to, the evolution of these systems. Based on the concept of production-living-ecology, resilience theory, and sustainable livelihood theory, a resilience assessment framework for tourism villages is constructed from the perspective of farmers in the following three dimensions: production resilience, living resilience and ecological resilience. Moreover, the comprehensive index method and an obstacle degree model are utilized to quantitatively measure and identify the resilience characteristics and obstacle factors of the tourism villages in Xiangxi Prefecture, China. The results reveal the following. (1) The three periods of the evolution of the resilience of tourism villages in Xiangxi Prefecture included the undeveloped tourism period, the normalized tourism period, and the COVID-19 disturbance period. Throughout these periods, tourism village resilience was characterized by a steady increase followed by a small decline, overall demonstrating a fluctuating upward trend. Rural tourism has had a positive effect on the long-term development of the rural territorial system, whereas the COVID-19 pandemic had a negative effect. (2) The tourism village resilience of farmers perspective with different livelihood types exhibits obvious heterogeneity; the tourism village resilience of tourism-led farmers is the highest, followed by that of part-time balanced farmers. Thus, these livelihood types are the main direction for the development and transformation of farmers with other livelihood types. (3) The obstacle factors to the evolution of the resilience of tourism villages in Xiangxi Prefecture exhibit a converging trend. In the three periods, the common obstacles to the evolution and development of tourism village resilience included the number of people participating in rural tourism, the tourism business area, and the area of farmland returned to forests.

## Introduction

The Chinese government introduced the Rural Revitalization Strategy in 2017. Rural revitalization is the road to national prosperity and national rejuvenation, the key and foundation of which is industrial revitalization^1^. The development of rural tourism in mountainous areas rich in tourism resources has become a scientific practice to broaden the channels for farmers to increase their income and enrich themselves, enhance the endogenous driving force of rural development, and realize rural revitalization^2,3^. Rural tourism is not only an important driving force and foothold for the implementation of the rural revitalization strategy, but also faces unprecedented development opportunities and challenges^4,5^. The predatory tourism development model and the influx of numerous non-agricultural industries and social factors into the rural territorial system have caused the structure of the production, living, and ecology space (PLES) within the system to suffer constant impacts. The contradiction of the PLES has become increasingly more obvious, and has been accompanied by changes in the livelihood types of farmers and the rupture of the balanced symbiotic relationship between human beings and nature. With the global outbreak of the COVID-19 pandemic in the early 2020s, the rural territorial system was also severely impacted, with significant implications for rural development. As the countryside is subjected to multiple natural and social perturbations and impacts, the allocation of resources, social culture, and the PLES of the rural territorial system have experienced deep-seated disturbances, and its instability and vulnerability have gradually become apparent^6–8^. Current issues facing the construction of the countryside include determining how to build a resilient rural territorial system capable of coping with and adapting to risks, completing transformation and reconstruction, and realizing the rural industry to improve the efficiency of farmers to increase their income. Rural tourism villages in ethnic areas can effectively enhance their core competitiveness by accurately grasping the characteristics of rural resilience^9^.

Resilience is a key issue in sustainability science^10^, and resilience-related research has broadly undergone different stages of engineering resilience, ecological resilience, and evolutionary resilience. Resilience was first applied to mechanical and other engineering fields, used to describe the material in the deformation under the action of external forces after the ability to recover^11^, which is the “engineering toughness”. Holling Holling then introduced the concept of resilience into the field of ecology, and pointed out that resilience can measure the stability of ecosystems, and the concept of resilience thus changed from “engineering resilience” to “ecological resilience”^12^. It follows that both engineering resilience and ecological resilience are based on the system being in a unique state of equilibrium. In the 1990s, Walker further introduced it into human social systems and developed the “adaptive cycle” model, which proposes that systems are in a dynamic process of continuous change and development, and that there is no single equilibrium state^13^. Evolutionary resilience refers to the ability of a system to return to its original or develop a new equilibrium after a disturbance and to achieve sustainable development^14^, emphasizing continuous adaptation, learning, and innovation, which makes the system stronger after a shock. Over the years, resilience research perspectives have expanded from ecological perspectives into disciplinary fields such as sociology^15^, urban and rural planning^16^ and rural geography^17^, and have become an important tool for understanding the sustainability of socio-ecological systems. The dynamic, adaptive, and nonlinear thinking emphasized by resilience theory has a natural fit and theoretical advantage in explaining the problems facing the sustainable development of rural territorial systems in the context of rural tourism and the COVID-19 disturbance^18,19^.

Rural resilience has been developed based on the theory of resilience; it is an inherent property of rural territorial systems, and is a direct manifestation of the ability of a rural area to develop sustainably^20^. The specific connotation refers to the ability of an rural territorial systems to resist and mitigate impacts when disturbed by internal and external factors, and to utilize economic, social, and environmental capital to interweave and develop multifunctionality and maintain the dynamic balance of society, the economy, culture, and ecology. This connotation emphasizes the characteristics of proactive adaptation, promptly responding to changes, and sustained development^21^. As a response to problems faced by the countryside, the study of rural resilience has received extensive attention from scholars and has become an important theoretical basis for understanding the interaction between the rural kernel system and the external environmental system^18^. At this stage, the research mainly involves the definition and evaluation of concepts related to rural resilience^22^, the identification of factors affecting rural resilience^23^, and resilient rural construction and governance^24^, among others. On top of clarifying the meaning of resilience, some scholars have elaborated on the positive role of resilience theory in urban construction and post-disaster reconstruction, such as pre-disaster early warning, and introduced resilience theory into rural construction, pointing out that resilience enhancement can help to strengthen the comprehensive strength of the countryside and promote the comprehensive revitalization of the countryside^18^. The evaluation of rural resilience has been studied in three main areas: disaster risk^25,26^, specific perturbations^27,28^ specific perturbations^26^. With regard to the identification of influencing factors, some scholars believe that the countryside, in the face of multiple shocks, the increase in its agricultural income and the development of non-farm enterprises have contributed to the increase in the level of economic resilience of the countryside^29^. It has also been shown through research that village resilience in Brazil is weakened by a reduction in landscape diversity and land use diversity, and that landscape homogenization makes villages less resilient^30^. In the area of resilient rural construction and resilient governance, some scholars have studied the use of digital network facilities to enhance village resilience, and have emphasized that government leadership and the endogenous motivation of farmers can enhance the ability of villages to cope with disruptions^31^. Conversely, few scholars have conducted longitudinal studies to describe the continuous changes of various factors and to explore the dynamic evolution of rural resilience from a developmental perspective. Related research has been conducted in tourist villages^32^, villages in deprived areas^33^, and Villages in minority areas^34^, among others. Scholars have constructed diversified rural resilience assessment frameworks for different research objects, among which the two most common are the “three-dimensional” rural resilience measurement model based on buffer capacity, self-organization capacity, and learning capacity^35^, and the “five-dimensional” rural resilience assessment framework based on livelihood capital^36^. In addition, a framework has also been constructed for the analysis of rural resilience based on social, economic, cultural, and political factors^37^. Presently, the production-living-ecology (PLE) concept, an important pillar of sustainable development, is widely recognized both domestically and internationally^38^. However, few scholars have constructed an analytical framework for rural resilience based on this concept that focuses on the sustainable development of the system, followed by a further analysis of rural resilience from the perspectives of production, living, and ecology. The research scale can be divided into the macroscale, mesoscale, and microscale. Macroscale studies focus on nations^23^, regions^39^and provinces^19^; mesoscale studies are generally conducted in municipalities^40^ and counties^32^; and microscale research mostly concerns communities^41^. However, rural resilience has rarely been investigated from the micro-perspective of rural households, the primary actors of rural activities. The main research methods include the comprehensive index method^23^, principal component analysis^42^, variable fuzzy recognition models^43^, the TOPSIS method^44^, the set pair analysis method^45^, and other quantitative resilience measurement methods, among which the comprehensive index method is the most widely used.

In summary, few scholars have analyzed in depth the dynamic evolution of tourism village resilience based on the PLE concept from the micro-perspective of farmers. Therefore, in this study, eight typical tourist villages in Xiangxi Prefecture, China, were selected as the study objects. Based on field research data and in combination with the PLE concept, resilience theory, and sustainable livelihood theory, a framework for the analysis of the resilience of tourist villages from the perspective of farmers, as well as an evaluation index system, were constructed. Moreover, the resilience of tourist villages against the background of rural tourism and the disturbance of the COVID-19 pandemic was explored. The goal of this research is to provide theoretical support and a case reference for promoting the sustainable development of tourism villages in other underdeveloped ethnic regions worldwide.

## Materials and methods

### Study area

**Fig. 1:**
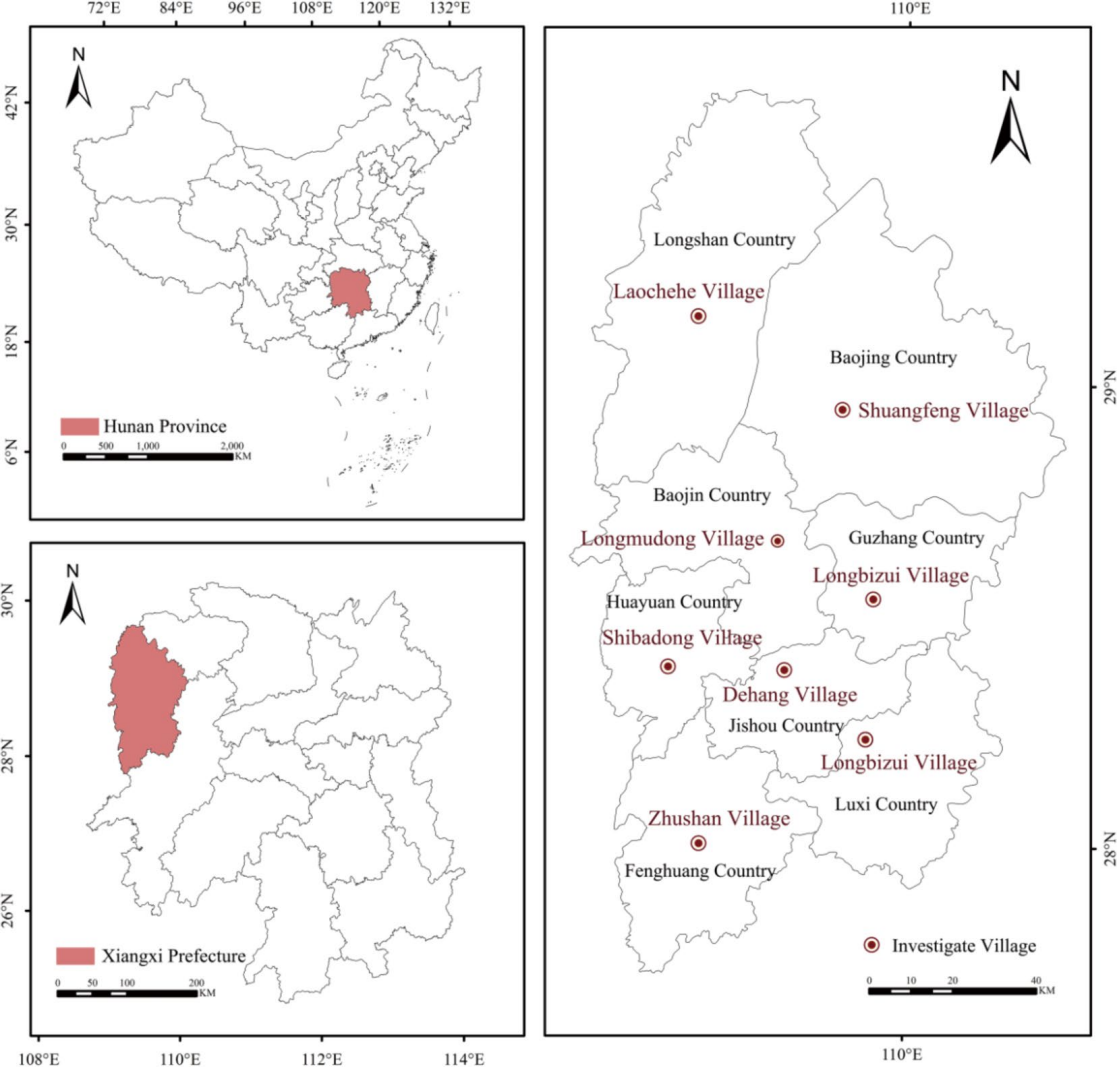
The location of the study area and study villages.

Xiangxi Tujia and Miao Autonomous Prefecture (herein referred to as Xiangxi Prefecture) is located in the core area of China’s Wuling Mountain area in northwestern Hunan Province, and is dominated by mountainous terrain with distinctive cultural characteristics and rich tourism resources (Fig. 1). It is both a multi-ethnic and multi-cultural integration of ethnic minority areas and contiguous underdeveloped areas. It is characterized by both the national biodiversity protection and soil and water conservation of key ecological functional areas, and is also one of China’s few cultural and ecological reserves. Xiangxi Prefecture is extremely rich in tourism-based resources, and rural tourism has had a significant impact on the rural socio-economic structure and the composition of farmers’ livelihoods. It has thus played a positive role in socio-economic development, consolidating the results of poverty alleviation, promoting regional development and ethnic integration, and realizing the rural revitalization strategy.

To deeply explore the impacts of rural tourism and COVID-19 on the resilience of tourism villages in Xiangxi Prefecture, the case villages were selected in consideration of their overall spatial distribution within Xiangxi Prefecture, their better social and economic benefits, their certain degree of popularity at the regional level, the deeper impact of rural tourism on these villages, and the certain degree of differentiation in the development of tourism.Specifically, we selected eight tourism-oriented villages, including Dehang Village, which are distributed evenly across the eight counties of Xiangxi Prefecture. Under limited research resources, these case villages not only cover the geographical and cultural diversity of Xiangxi Prefecture but also effectively represent its regional characteristics. (Fig. 1). In addition, the data obtained from the research included material, financial, social, ecological, and other types of data that convey rich and comprehensive information. The data reflect the resilience of tourism villages in Xiangxi Prefecture based on the livelihoods of farmers, and the research samples have strong typicality and representativeness.

### Data sources

The research data were mainly obtained from semi-structured interviews and questionnaires. First, relevant information about the society, tourism economy, tourism industry, and ecological environment of Xiangxi Prefecture was obtained via a data review to understand the basic situation of tourism development and ecological protection in the prefecture. As a complex, dynamic, and comprehensive composite system, the development of rural tourism has significant staged characteristics. In order to better identify the resilience evolution characteristics of tourism villages In order to better identify the characteristics of rural tourism and the evolution of rural resilience in Xiangxi Prefecture under the new crown epidemic, this paper selects to obtain the basic livelihood characteristics data of rural households in the case villages of the undeveloped tourism period, the tourism normalization period, and the COVID-19 disturbance period.The research team conducted a pre-survey in December 3 to 15 ,2022 in the three case villages of Dehang, Zhushan, and Laochehe, and, based on the results, the questionnaires and interview outlines were revised and improved. The formal research was then carried out in July and August 2023. In-depth interviews, were conducted with village chiefs or other key members of the village committee and typical farmers to gain a comprehensive understanding of the social, economic, ecological, and livelihood conditions of the villages. This is used to delineate the time points between the undeveloped tourism period and the tourism normalization period, and the period of COVID-19 disruption is the period of COVID-19 outbreaks.This was followed by a household questionnaire survey distributed using the quota sampling method. Based on the Participatory Rural Appraisal methodology and farmers’ recollections, each farmer was interviewed about the actual livelihood of the undeveloped tourism period, the tourism normalization period, and the COVID-19 disturbance period, each lasting about 30–60 min. In this way, the basic livelihood characteristics of rural households in three periods were obtained. A total of 312 questionnaires were distributed, and after removing invalid questionnaires and those with abnormal data, a total of 303 valid questionnaires were ultimately recovered, reflecting a validity rate of 97.12% (Table 1).

**Table 1:** The basic characteristics of the sample of farmers and the distribution of questionnaires in Xiangxi Prefecture.

Item	Form	Number	Proportion	Item	Form	Number	Proportion
Gender	Male	154	50.83	Nation	Tujia	124	40.92
					Miao	173	57.10
	Female	149	49.17		Han	6	1.98
Age (years)	≤ 18	2	0.66	Annual household income (RMB)	≤ 1	6	1.98
	19–25	5	1.65		1.0001–5.0000	42	13.87
	26–40	49	16.18		5.0001–10.0000	111	36.63
	41–60	132	43.56		10.0001–25.0000	138	45.54
	≥ 61	115	37.95		≥ 25.0001	6	1.98
Village	Shuangfeng	26	8.58	Education level	Primary school or below	190	62.71
	Dehang	41	13.53				
	Zhushan	39	12.87		Junior high school	85	28.05
	Xinzhaiping	37	12.21				
	Longbizui	42	13.87		Senior high school	21	6.93
	Shibadong	38	12.54				
	Longmudong	40	13.20		College degree or above	7	2.31
	Laochehe	40	13.20				

## Analytical framework and indicator system

### Analytical framework

An rural territorial systems is a composite system with a certain function and structure within a specific rural area, and is composed of interactions among the natural environment, resource endowment, economic base, human resources, cultural customs, and other elements^38^. The PLES is an important component of an rural territorial systems, and is essentially a complex system of interwoven human production, living, and ecological behavioral activities^46^. The PLE concept was developed based on PLES research, and aims to realize the coordinated and sustainable development of the production, living, and ecology of a system via the creation of intensive and efficient production, appropriate living conditions, and green ecological development. This is consistent with the characteristics of resilience theory and sustainable livelihood theory for the realization of sustainable development. The countryside is the most solid foundation for the high-quality development of China’s economy and the great rejuvenation of the Chinese nation, and the enhancement of rural resilience is necessary for its sustainable development. To better explore the characteristics of the evolution of tourism village resilience in Xiangxi Prefecture, in combination with existing studies^47–49^ and drawing on the concept of PLE, resilience theory, and sustainable livelihood theory, tourism village resilience from the perspective of farmers is defined as follows: under exogenous shocks and endogenous perturbations in the rural territorial systems, farmers continuously adjust their own livelihood structure from the production, living, and ecological levels to buffer, resist, and adapt to disaster risks, so that the system continues to develop toward a new dynamic equilibrium.

**Fig. 2:**
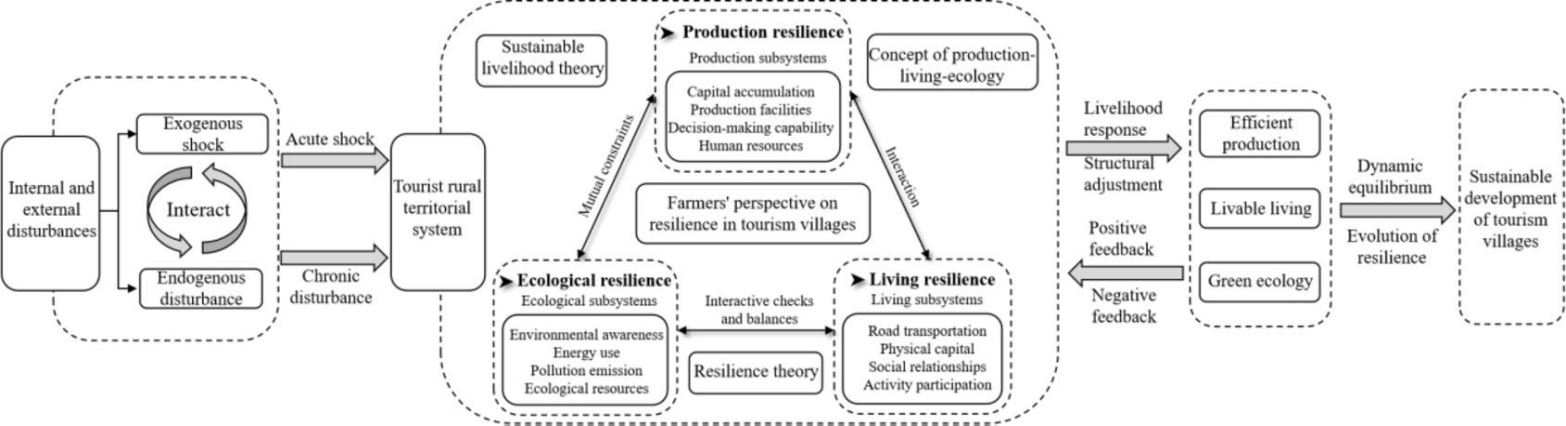
The framework for analyzing the resilience of tourist villages from the perspective of farmers.

When the rural territorial systems is subjected to internal and external chronic perturbations and acute shocks, farming families can use their livelihood capital to buffer and respond to the management of their livelihoods from the three aspects of production, living, and ecology. This can promote the restructuring of the structure and function of the rural territorial systems, thus realizing the dynamic balance of efficient production, living, and ecological greening, and ultimately promoting the evolution of tourism village resilience to realize the sustainable development of the tourism village (Fig. 2). Based on the PLE concept, tourism village resilience includes the three subsystems of production resilience, living resilience, and ecological resilience, and the behavioral activities of farmers are the medium of resilience in the rural territorial systems. The three subsystems synergistically interact with each other via interactive checks and balances, thus complementing each other and promoting the continuous development and evolution of tourism village resilience.

### Indicator system

Villages and farmers are symbiotic; tourism village resilience is the macro-expression of farmers’ livelihoods, farmers’ livelihoods are the micro-manifestation of tourism village resilience, and a change in one is bound to cause a change in the other. Farmers are the main body and center of interest of rural tourism activities, and the impacts of their livelihood capital and livelihood activities on the rural territorial systems reflect the resilience of the tourism village. The stronger the resilience, the stronger the promotion of the development of the rural territorial systems via the livelihood capital and livelihood activities of farmers from the three aspects of production, living, and ecology, and the stronger the ability of the rural territorial systems to withstand external shocks and internal disturbances. Based on the analytical framework of tourism village resilience and following the principles of scientificity, operability, and comprehensiveness, a comprehensive evaluation index system is constructed from the three aspects of production resilience, living resilience, and ecological resilience (Table 2). Among them, Production resilience in this context refers to the ability of farmers to utilize their resource endowment to obtain better production conditions and services to achieve sustainable production activities; this is characterized by indicators including the number of people participating in rural tourism, the diversity of livelihoods, and the tourism business area. Living resilience is the ability of farmers to maintain or improve their living conditions based on financial, material, social, and other capital; this is characterized by the type of road leading to the household, the total household fixed assets, and the annual expenditure on favors, among other factors. Finally, Ecological resilience is the ability to provide farmers with a means of production and living and to maintain ecological sustainability; this is characterized by indicators such as the area of farmland returned to forests, ecological and environmental protection awareness, and the cleanliness of travel modes.

**Table 2:** The indicator system for measuring the resilience of tourist villages in Xiangxi Prefecture from the perspective of farmers.

Target level	Dimension layer	Indicator layer	Variable assignment	Weight
	Production resilience	Number of participants in rural tourism, C_1_	1 = 0 people; 2 = 1 people; 3 = 2 people; 4 = 3 people; 5 = 4 or more people (+)	0.1463
		Tourism business area, C_2_	1 = 0–10 m^2^; 2 = 10–15 m^2^; 3 = 15–30 m^2^; 4 = 30–40 m^2^; 5 = above 40 m^2^ (+)	0.1439
		Livelihood diversity, C_3_	1 = 1 type; 2 = 2 types; 3 = 3 types; 4 = 4 types; 5 = 4 or more types (+)	0.0273
		Annual household income, C_4_	1 = less than 30,000 RMB; 2 = 30,000–65,000 RMB; 3 = 65,000–100,000 RMB; 4 = 100,000–180,000 RMB; 5 = above 180,000 RMB (+)	0.0121
		Loan amount, C_5_	1 = less than 10,000 RMB; 2 = 10,000–20,000 RMB; 3 = 20,000–40,000 RMB; 4 = 40,000–60,000 RMB; 5 = above 60,000 RMB (-)	0.0038
Tourism village resilience	Living resilience	Type of road leading to the home, C_6_	1 = Unhardened road not open to traffic; 2 = Hardened road accessible to motorcycles; 3 = Unhardened road accessible to cars and buses; 4 = Hardened road accessible to cars; 5 = Hardened road accessible to cars and buses (+)	0.0557
		Total household fixed assets, C_7_	1 = 0–20,000 RMB; 2 = 20,000–40,000 RMB; 3 = 40,000–80,000 RMB; 4 = 80,000–140,000 RMB; 5 = above 140,000 RMB (+)	0.0759
		Annual expenditure on favors, C_8_	1 = 0–1,600 RMB; 2 = 1,600-4,000 RMB; 3 = 4,000–1,3000 RMB; 4 = 1,3000-30,000 RMB; 5 = above 30,000 RMB (+)	0.0479
		Degree of interaction with powerful people and large families, C_9_	1 = None; 2 = Occasionally; 3 = Sometimes; 4 = Often; 5 = Frequently (+)	0.0468
		Yearly participation in ethnic minority cultural activities, C_10_	1 = 0 times; 2 = 1 time; 3 = 2 times; 4 = 3 times; 5 = 4 or more times (+)	0.1070
	Ecological resilience	Ecological awareness, C_11_	1 = Extremely poor; 2 = Poor; 3 = Fair; 4 = Good; 5 = Extremely good (+)	0.0637
		Area of farmland returned to forests, C_12_	1 = Less than 0.02 hm^2^; 2 = 0.02–0.047 hm^2^; 3 = 0.047–0.08 hm^2^; 4 = 0.08–0.13 hm^2^; 5 = above 0.13 hm^2^ (+)	0.1701
		Travel mode cleanliness, C_13_	1 = Car; 2 = Motorcycle; 3 = Bus; 4 = Electric vehicle; 5 = Walking (+)	0.0570
		Annual pesticide and fertilizer costs, C_14_	1 = 0–180 RMB; 2 = 180–650 RMB; 3 = 650–1300 RMB; 4 = 1300–3000 RMB; 5 = above 3000 RMB (-)	0.0110
		Monthly energy costs, C_15_	1 = 0–45 RMB; 2 = 45–90 RMB; 3 = 90–160 RMB; 4 = 160–300 RMB; 5 = above 300 RMB (-)	0.0316

## Methods

### Comprehensive index method

In the context of this research, tourism village resilience is understood as a composite of the three dimensions of production resilience, living resilience and ecological resilience, which can be measured by the comprehensive index method. The entropy value method is first used to establish the index weights. Drawing on existing research and considering that all dimensions are equally important for the enhancement of tourism village resilience (as determined by expert consultation), the dimensions were determined to have equal weights. The formula is as follows^50^:

1 $${P}_{I}=\sum_{j=1}^{5}{\omega\:}_{j}{Y}_{ij}$$

2 $${L}_{I}=\sum_{j=6}^{10}{\omega\:}_{j}{Y}_{ij}$$

3 $${E}_{I}=\sum_{j=11}^{15}{\omega\:}_{j}{Y}_{ij}$$

4 $$\:{\:R}_{I=}{{W}_{P}P}_{I}+{{W}_{L}L}_{I}+{W}_{E}{E}_{I}$$

where *R*_*I*_ denotes tourism village resilience from the perspective of farmers; *P*_*I*_, *L*_*I*_, and *E*_*I*_ denote the production resilience index, living resilience index, and ecological resilience index, respectively; *W*_*p*_, *W*_*L*_, and *W*_*E*_ denote the weights of the three dimensions of production resilience, living resilience, and ecological resilience, respectively; *ωj* denotes the stratum weight of the *j*th index; and *Y*_*ij*_ represents the standardized value of the *i*th index of the *j*th research unit.

### Obstacle degree model

To further identify the obstacles to the resilience evolution of tourism villages, the obstacle degree model is applied to analyze the impact of each evaluation index on the resilience evolution of tourism villages. The formula is as follows^51^:

5 $$\:{P}_{ij}={1} - {Y}_{ij}$$

6 $${I}_{j}={P}_{ij} {\omega}_{j}/{\sum}_{j=1}^{15}{P}_{ij}\; {\omega}_{j} {\times {100} \%}$$

where *P*_*ij*_ represents the indicator deviation, which indicates the gap between a single indicator and the optimal target value; *I*_*j*_ represents impediment, which indicates the degree to which the *j*th indicator is an impediment to tourism village resilience; and *ωj* represents the weight of a single factor for the overall objective, or the weight of the *j*th index relative to tourism village resilience.

### Ethical approval

This study received approval from the Research Ethics Committee of Jishou University, Zhagjiajie, Hunan, China (JSDX-2022-002) on November 26, 2022. The study was performed in accordance with the Declaration of Helsinki. Informed consent was obtained from all subjects .The questionnaires were anonymized to protect their privacy. Participants were also given the option to decline participation in the survey if they chose to do so.

## Results

### Characteristics of the evolution of the resilience of tourist villages in Xiangxi Prefecture

Equations (1)-(4) were applied to measure the resilience of tourism villages in Xiangxi Prefecture from the perspective of farmers. By referring to other classification standards for tourism village resilience^52^and in combination with the characteristics of the village resilience data of the eight case villages in Xiangxi Prefecture, village resilience was classified into three levels of low, medium, and high via the natural breakpoint method^53^ (Table 3).

**Table 3:** The discriminative criteria for the evaluation of the resilience of tourism villages in Xiangxi Prefecture.

Evaluation	Rating	Index values
Production resilience	Low resilience	[0.0000–0.0165]
	Medium resilience	[0.0166–0.1102]
	High resilience	[0.1103–0.2785]
Living resilience	Low resilience	[0.0000–0.0995]
	Medium resilience	[0.0996–0.1733]
	High resilience	[0.1734–0.3097]
Ecological resilience	Low resilience	[0.0161–0.0815]
	Medium resilience	[0.0816–0.2129]
	High resilience	[0.2130–0.2937]
Tourism village resilience	Low resilience	[0.0784–0.2690]
	Medium resilience	[0.2691–0.4012]
	High resilience	[0.4013–0.7273]

**Fig. 3:**
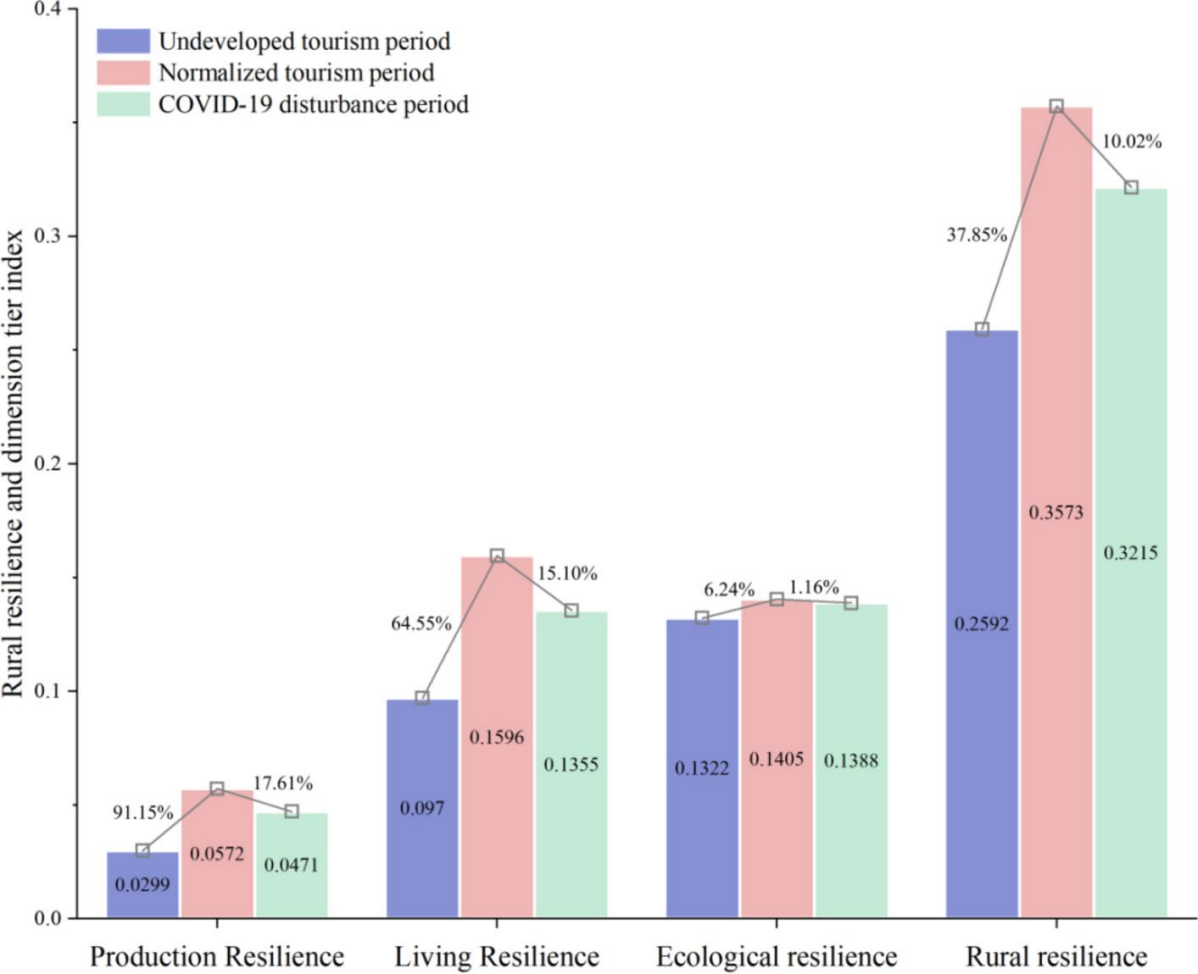
The rural resilience and the resilience level of each dimension of tourism villages.

In order to deeply explore the evolutionary characteristics of tourism village in Xiangxi Prefecture under rural tourism and COVID-19, we choose to analyze the three periods from the undeveloped tourism period, the tourism normalization period, and the COVID-19 disturbance period.The results show that, from the perspective of farmers, the tourism village resilience index of Xiangxi Prefecture in the undeveloped tourism period, the tourism normalization period, and the COVID-19 disturbance period were 0.2592 (low), 0.3573 (medium), and 0.3215 (medium), respectively. Overall, the tourism village resilience of Xiangxi Prefecture in the study period exhibited a fluctuating upward trend, and the growth rate of the tourism village resilience index was 24.04% (Fig. 3). During the undeveloped tourism period, the resilience of tourism villages in Xiangxi Prefecture was at a low level; during the period of normalized tourism, the tourism village resilience increased significantly to a medium level; and during the period of COVID-19 disturbance, the tourism village resilience decreased only slightly, and still remained at a medium level. This shows that since the influx of tourism as an external disturbance to the rural territorial system, the livelihood level of farmers has increased significantly, and the production mode, living standards, and ecological environment have all been enhanced. Moreover, the internal functional structure of the rural territorial systems has tended to be more stable, which mitigated the impact of the COVID-19 disturbance. It is evident that the development of tourism in the countryside has had a positive effect that is conducive to the improvement of the livelihood level of farmers, the intensive use of rural resources, and ecological environmental protection; thus, it has effectively enhanced the resilience of the countryside, reflecting a path toward sustainable development.

in Xiangxi Prefecture in three periods.

**Fig. 4:**
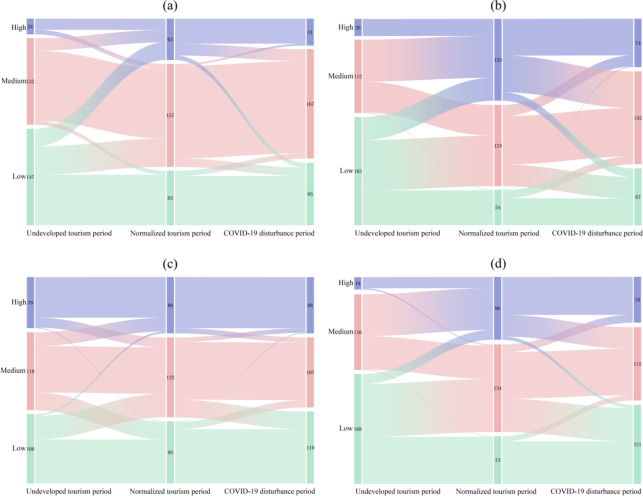
The changes in the resilience of tourism villages in Xiangxi Prefecture and the resilience level of each dimension in the three periods: (**a**) Production resilience; (**b**) Living resilience; (**c**) Ecological resilience; (d)Tourism village resilience.

Specifically, from the undeveloped tourism period to the normalized tourism period, the resilience of tourism villages in Xiangxi Prefecture increased by 37.85%. Production resilience nearly doubled with an increase of 91.15%, followed by the increases of living resilience (64.55%) and ecological resilience (6.24%). Moreover, heterogeneity was found in the evolution of tourism village resilience from the farmers’ perspective; focusing on the transition from low and medium grade tourism village resilience farmers to medium and high grades (Fig. 4). In terms of the dimensions of tourism village resilience, production resilience was found to have increased significantly over the study period, which is reflected in the shift of farmers with low grade production resilience to medium and high grade. After the influx of tourism into the rural territorial systems, farmers gave full play to their subjective initiative and increased their enthusiasm for tourism participation, the main impetus for which was the increase in family tourism participation. The resilience of tourism villages has been significantly improved, and many farmers with medium resilience, as well as some with low resilience, have advanced to high resilience, and the number of high-resilience farmers has increased dramatically. The reason for this is that tourism development has greatly improved the infrastructure and public service facilities in the villages, which has facilitated the living and production activities of farmers and improved their living standards. Furthermore, the ecological resilience of villages has increased slightly, and the ecological resilience level of many farmers has improved only to the adjacent level. Additionally, the number of farmers between different levels has changed in a dynamic and balanced way, and the ecological resilience level of only a few farmers has changed due to the disturbance of endogenous or external factors (Fig. 4). This indicates that tourism has had a certain positive effect on the rural territorial systems, which is conducive to improving the stability of the internal structure of the system, significantly improving the sustainability of the livelihood of farmers, and thus enhancing the resilience of the countryside.

From the normalized tourism period to the COVID-19 disturbance period, the resilience of tourism villages in Xiangxi Prefecture decreased slightly by 10.02%. Among the three dimensions, production resilience and living resilience decreased to a slightly greater extent (17.61% and 15.10%, respectively), while ecological resilience fluctuated less (1.16%) (Fig. 3). The evolution of tourism village resilience also exhibited heterogeneity; this was mainly manifested as the transformation of famers with medium and high grade tourism village resilience to medium and low tourism village resilience.

From the perspective of the dimensions of tourism village resilience, the rural production resilience index declined from 0.0572 to 0.0471 during the study period, reflecting the largest decrease among the three dimensions. The distribution of the production resilience grades indicates that the number of medium-grade production resilience farmers was the largest, and the resilience grades of some medium-grade production resilience farmers and high-grade production resilience farmers were downward to the neighboring grades, while the production resilience level increased dramatically of a small number of farmers. Under the exogenous shock of the COVID-19 pandemic, rural tourism suffered from reduced employment opportunities for farmers and reduced tourism participation, thus decreasing PR.

Furthermore, the rural living resilience index fell from 0.1596 to 0.1355; this is reflected in the largest number of farmers with medium living resilience. The number of farmers with high living resilience decreased sharply, and the living resilience of these households decreased to the medium and low grades. However, the living resilience of some farmers did increase to higher grades. The livelihood capital accumulation of farmers after tourism development was used to withstand the mitigation of the COVID-19 shock, and the living standards were reduced, resulting in the reverse evolution of living resilience levels.

Furthermore, the rural ecological resilience index decreased from 0.1405 to 0.1388, the smallest decrease among the three dimensions of tourism village resilience. The number of households with high ecological resilience was dynamically conserved, while households with medium ecological resilience experienced a decrease to the lower ecological resilience level (Fig. 4). During the COVID-19 disturbance period, the home energy consumption of farmers increased and their awareness of ecological conservation weakened; however, their tourism and agricultural production activities decreased, and the overall ecological disadvantages slightly outweighed the ecological advantages.

These results show that COVID-19 had a negative impact on the rural territorial systems, but the capital accumulation and livelihood transformation of farmers due to their participation in tourism moderated the negative impact. Thus, the stability of the rural territorial systems structure was maintained, and its resilience decreased only slightly under the strong shock of the COVID-19 pandemic.

### Classification and characterization of the livelihood types of farmers

According to existing research^54^ and the development of Xiangxi Prefecture, the livelihood types of farmers were divided into four categories according to the proportion of household income for which the livelihood accounts. These categories include labor-led, tourism-led, agriculture-led, and part-time balanced livelihoods (Table 4).

**Table 4:** The criteria, sample size, and percentage of farmers with different livelihood types.

Type	Division basis	Quantity					
		Undeveloped tourism period	Proportion	Normalized tourism period	Proportion	COVID-19 disturbance period	Proportion
Tourism-led	Proportion of tourism income ≥ 60%	17	5.61	50	16.5	28	9.24
Agriculture-led	Proportion of agricultural income ≥ 60%	35	11.55	19	6.27	14	4.62
Labor-led	Proportion of working income ≥ 60%	218	71.95	178	58.75	214	70.63
Part-time balanced	Balanced and diversified sources of income	33	10.89	56	18.48	47	15.51

1. Tourism-led. Farmers’ livelihood activities are significantly affected by tourism development, and are mostly distributed along tourist transportation routes and in the key development areas of villages. These areas enjoy better transportation conditions and higher tourist flow, which are associated with obvious advantages for tourism operations. Such farmers mostly choose to operate bed and breakfasts, restaurants, and tourist souvenir stores. The combination of their livelihood types is mainly “tourism business + short-term labor.”
2. Agriculture-led. Livelihood activities are greatly affected by the natural environment and transportation conditions, and it is more difficult to satisfy a family’s living needs simply by engaging in agricultural production activities. Therefore, some farmers also engage in short-term local labor, such as temporary odd jobs, small construction jobs, and part-time agricultural work. The combination of their livelihood types is mainly “farming + short-term labor.”
3. Labor-led. Family laborers are mainly engaged in labor activities, working year-round in developed coastal areas and in counties and cities around the family, and basically do not participate in tourism activities due to insufficient capital accumulation or lack of optimism about rural tourism development.The combination of their livelihood types is mainly“permanent labor + short-term labor + farming”.
4. Part-time balanced. Family livelihood activities are based on activities such as running kiosks and mobile stalls in the village, and the scale is relatively small. Individual laborers are engaged in jobs such as security guards and cleaning staff in scenic spots, and waiters in restaurants or lodgings. Such residents carry out agricultural production and short-term labor, and the combination of their livelihood types is mainly “tourism operations + short-term labor + farming.”

### Characteristics of the evolution of the resilience of tourist villages in Xiangxi Prefecture from the perspective of farmers with different livelihood types

**Fig. 5:**
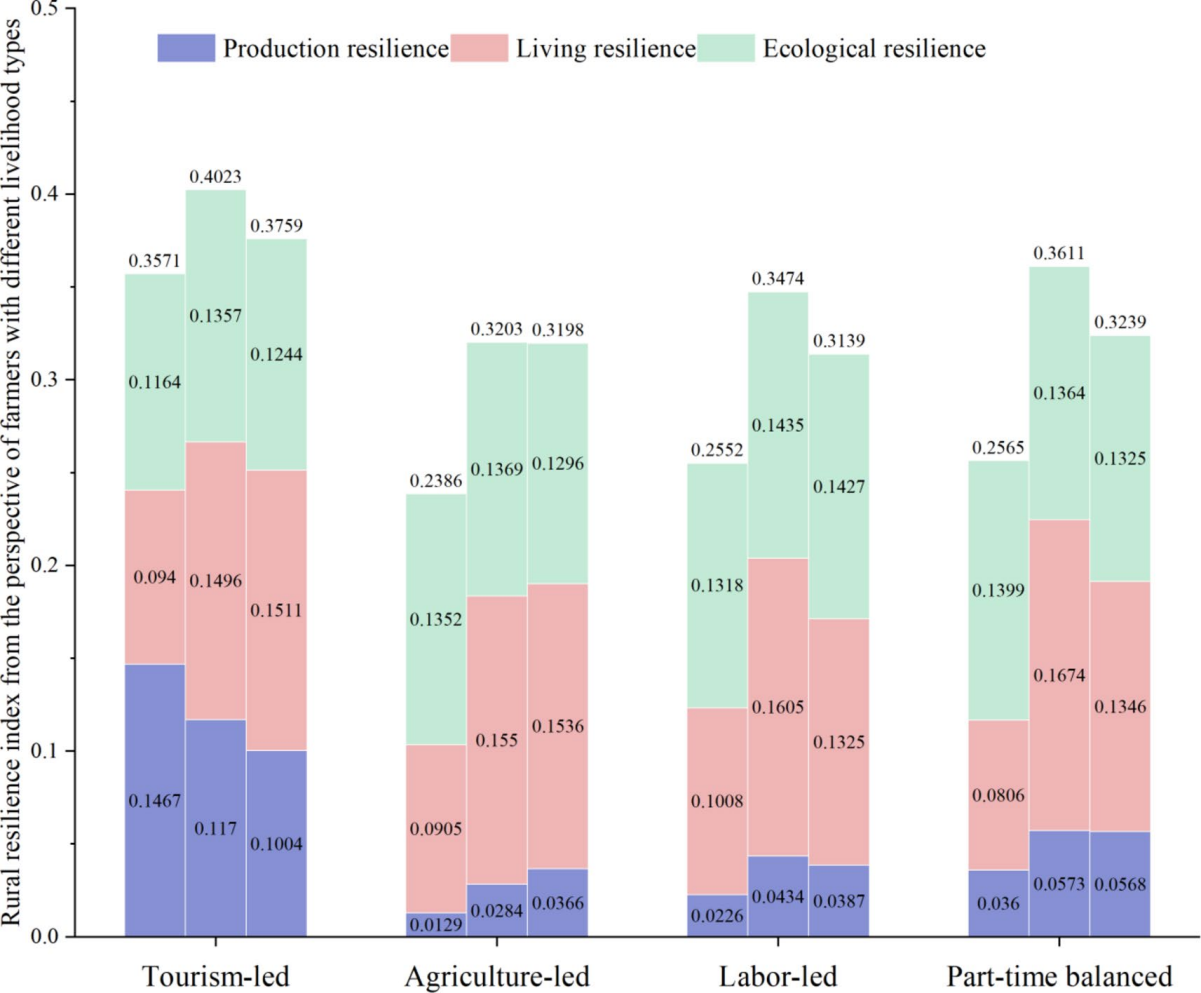
The tourism village resilience index from the perspective of farmers with different livelihood types over three periods.

**Fig. 6:**
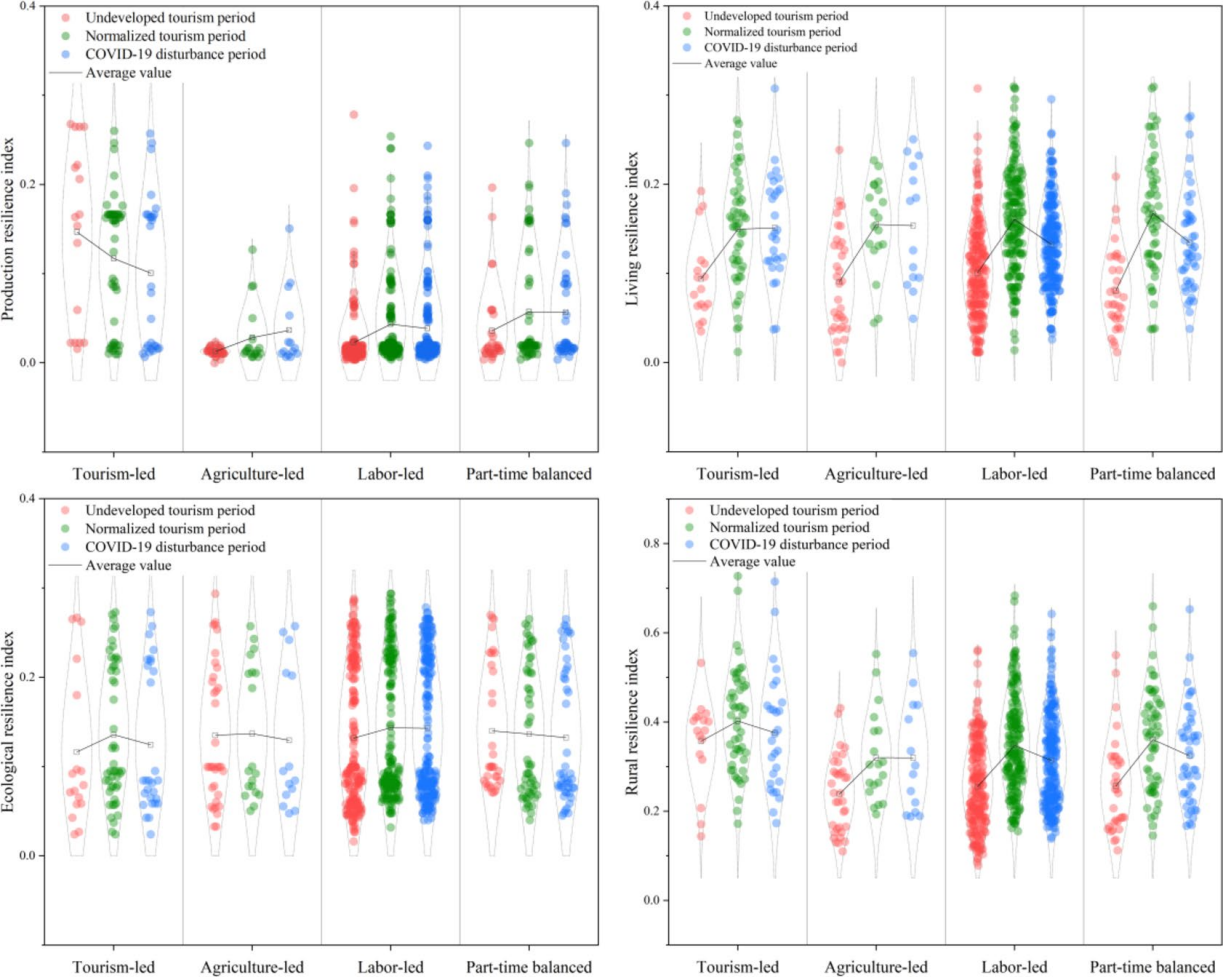
The tourism village resilience and the resilience level of each dimension from the perspective of farmers with different livelihood types in the three sub-periods.

The tourism village resilience evolution characteristics of tourism villages from the perspective of farmers with different livelihood types are subject to the combined effects of many endogenous and exogenous factors, and there may exist a certain degree of variability. To further analyze these differences, the level of resilience of tourism villages from this perspective was determined (Fig. 5). On the whole, the tourism village resilience of farmers with different livelihood types, from high to low, was found to be as follows: tourism-led, part-time balanced, labor-led, and agriculture-led. The highest value of the tourism village resilience index was found to reach 0.7273, while the lowest value was only 0.1121, reflecting a significant difference (Fig. 6).

Differences in the household structure, agricultural production methods, livelihood activities, and household capital accumulation among farmers with different livelihood types affect their performance in the three dimensions of tourism village resilience (Fig. 6). A deeper analysis reveals the following.

1. During the three tourism periods, the tourism village resilience scores of tourism-led farmers were respectively 0.3571 (medium), 0.4023 (high), and 0.3579 (medium), reflecting the highest values among the four livelihood types. These farmers were the only group that reached a high level of tourism village resilience. Furthermore, over time, the tourism village resilience level and the number of farmers first increased and then decreased, and the internal differentiation showed a medium level of agglomeration. The internal differentiation of ecological resilience was the same as those of the farmers characterized by other livelihood types, with few median values and many extreme values. This is due to the differences in the ways in which farmers participate in tourism. Some farmers engage in tourism services such as catering and lodging; these farmers consume more energy, travel in a less clean way, and cause more damage to the ecological environment. On the contrary, engaging in tourism services such as guided tours and stall operations has less ecological impact and is more conducive to the sustainable development of the rural territorial systems.
2. The resilience of agriculture-led farmers was the lowest over the study period, with respective tourism village resilience indices of 0.2386 (low), 0.3203 (medium), and 0.3198 (medium) for the three sub-periods. The number of farmers decreased period by period, but the resilience value fluctuated the least under the COVID-19 disturbance, and the internal differentiation revealed low agglomeration. To date, the economic status of agriculture has declined, and due to geographic constraints, farmers generally do not tend to use low-efficiency agricultural income as the main source of household income.
3. The tourism village resilience scores of labor-led farmers in the three sub-periods were respectively 0.2552 (low), 0.3474 (medium), and 0.3139 (medium), exhibiting the most widespread set of values with an olive-shaped internal differentiation. The tourism village resilience of these farmers fluctuated due to the positive effect of rural tourism development and the negative effect of the COVID-19 disturbance. Compared with those of agriculture-led farmers, the rural production resilience and ecological resilience dimensions of labor-led farmers were superior, but the internal differentiation of production resilience remained clustered with low values. Labor-led farmers are characterized by a high income and low threshold, and rural tourism has remained the primary choice for most farmers since the initiation of tourism development. However, the instability of labor-led farmers leads to the vulnerability of their livelihoods; thus, their level of tourism village resilience was found to be average.
4. From the perspective of part-time balanced farmers, the tourism village resilience indices in the three sub-periods were respectively. 0.2565 (low), 0.3611 (medium), and 0.3239 (medium). The overall tourism village resilience level of these farmers was found to be high, second only to that of tourism-led farmers, and with a more even internal differentiation. The tourism village resilience development of these farmers was found to be more balanced, and, compared with labor-led farmers, their rural production resilience was better, while their living resilience and ecological resilience had distinctive characteristics. The livelihood activities of part-time balanced farmers are diversified and well-founded, and their living resilience, like that of agriculture-led farmers, has risen despite the COVID-19 shock.

Based on the analysis of the tourism village resilience results from the perspectives of four different livelihood types, rural tourism in the rural territorial systems and the COVID-19 pandemic were found to have important impacts on the livelihood mode of farmers. By giving full play to their subjective initiative and relying on diversified livelihoods to adapt to changes and resist risks, farmers have realized the sustainability of their livelihoods, which has ultimately led to the sustainable development of the rural territorial systems. Among the three dimensions, living resilience and ecological resilience were found to have the most significant impacts on the resilience of tourism villages from the perspective of farmers; they are the dominant forces in changing the livelihood strategies of farmers and enhancing the resilience of tourism villages. Large differences were found in the resilience of tourism villages from the perspective of different livelihood types, with the highest resilience being that of tourism-dominant farmers, followed by part-time balanced farmers. Thus, these two livelihood types are the main directions for the development and transformation of the livelihoods of the remaining farmers, and contribute to the resilience enhancement and the sustainable and balanced development of the rural territorial systems.

### Obstacles to the evolution of resilience in tourist villages

Equations (5)-(6) were used to calculate the degree to which each obstacle hinders the evolution of the resilience of tourism villages. An obstacle with a cumulative contribution rate of more than 50% was considered to be the dominant factor [3], and the cumulative obstacle degree of the top 5 obstacle factors ranked by the four types of farmers is more than 70%, the top 5 obstacles were selected as the main factors affecting the resilience of tourism villages. According to the results based on the analysis of 303 farmers, the obstacles to tourism village resilience exhibited a convergence trend. Moreover, three common obstacles to tourism village resilience in the three periods were identified as follows: the number of people who participate in rural tourism (C_1_), the tourism business area (C_2_), and the area of farmland returned to forests (C_12_) (Table 5). Among them, C_1_ and C_2_ are indicators in the production resilience dimension, while C_12_ falls within the ecological resilience dimension. The obstacle degrees of C_1_ and C_2_ exhibited fluctuating growth trends over the three periods, with the values of C_1_ being 14.49%, 24.22%, and 20.61%, respectively, and the values of C_12_ being 10.41%, 17.963%, and 15.37%, respectively. The obstacle degree of C_2_ exhibited a fluctuating decreasing trend over the study period, with respective values of 21%, 14.66%, and 16.57% in each sub-period.

**Table 5:** The degree to which Obstacles hinder the resilience evolution of tourism villages.

Phase	Factor	Handicap	Order of obstacles	Cumulative obstacle degree
Undeveloped tourism period	Tourism business area, C_2_	21.00	1	21.00
	Ecological awareness, C_11_	18.02	2	39.02
	Number of participants in rural tourism, C_1_	14.49	3	53.51
	Total household fixed assets, C_7_	11.79	4	65.30
	Area of farmland returned to forests, C_12_	10.41	5	75.71
Normalized tourism period	Number of participants in rural tourism, C_1_	24.22	1	24.22
	Area of farmland returned to forests, C_12_	17.96	2	42.18
	Tourism business area, C_2_	14.66	3	56.84
	Total household fixed assets, C_7_	7.49	4	64.33
	Travel mode cleanliness, C_13_	6.59	5	70.92
COVID-19 disturbance period	Number of participants in rural tourism, C_1_	20.61	1	20.61
	Participation in folk cultural activities per year, C_10_	18.20	2	38.81
	Tourism business area, C_2_	16.57	3	55.38
	Area of farmland returned to forests, C_12_	15.37	4	70.75
	Travel mode cleanliness, C_13_	8.78	5	79.52

During the period of undeveloped tourism, the tourism business area (C_2_), the number of people participating in rural tourism (C_1_), and the total household fixed assets (C_7_) were the primary obstacles to the resilience of tourism villages from the perspective of farmers, and were respectively the first, third, and fourth most impactful obstacles. Before the countryside was formally developed for tourism, most farmers were engaged in agricultural production activities or go out to work, while very few took advantage of the transportation location to engage in low-technology-cost store operations in the family house to participate in tourism. Thus, similar to the tourism business area, the labor input at the farm household level and the physical capital input will change the livelihood strategy of farmers, and diversified livelihoods will significantly affect the rural territorial systems. Ecological awareness (C_11_) and the area of farmland returned to forests (C_12_) were respectively the second and fifth most impactful obstacles. The policy of returning farmland to forests was implemented in 2003, and farmers responded positively to the policy; it is a perfect ecological background resource that directly affects the ecological resilience of the countryside. Before rural tourism enters an rural territorial systems, farmers have relatively few opportunities to communicate with the outside world and exchange information, and their behavioral cognition and ecological and environmental protection awareness are slightly weak. Thus, the livelihood activities they carry out may have a certain impact on the ecology and other aspects of the rural territorial systems.

During the period of tourism normalization, the number of participants in rural tourism (C_1_) emerged as the primary obstacle to the evolution of tourism village resilience from the perspective of farmers. Moreover, the influence of the tourism business area (C_2_) decreased to the third major obstacle, and the total household fixed assets (C_7_) remained the fourth major obstacle. The entry of tourism factors into the rural territorial systems breaks the traditional balanced symbiotic relationship between man and nature. It provides farmers with a diversified choice of livelihood paths outside of traditional agriculture, thus increasing the enthusiasm of farmers to participate in tourism and operate stores, lodging inns, farms, and stalls, or to work as tour guides, scenic area staff, and other personnel directly or indirectly involved in rural tourism. The amount of livelihood capital reserves and the livelihood combination patterns in this sub-period underwent significant changes, and livelihood activities have positively contributed to the evolution of tourism village resilience. The area of farmland returned to forests (C_12_) jumped to the second largest obstacle to the tourism villageresilience of tourism, and the cleanliness of the travel mode (C_13_) replaced C_11_ as one of the main obstacles. The participation of farmers in tourism increases their communication opportunities with the outside world, improves their ability to obtain information, and gradually increases their ecological awareness. Consequently, farmers respond more positively to national ecological policies, adopt more scientific and reasonable livelihood behaviors, and reduce the impact on the ecological environment, which is conducive to the sustainability of the rural territorial systems.

In the COVID-19 disturbance period, the first and third most impactful obstacles to the resilience of tourism villages were consistent with those in the period of tourism normalization, i.e., the number of people participating in rural tourism (C_1_) and the tourism business area (C_2_), respectively. The second major obstacle was the yearly participation in folk culture activities (C_10_). Under the impact of the COVID-19 pandemic, rural tourism was significantly affected and the livelihood activities and behaviors of farmers, as the most central actors in rural tourism, were directly impacted. This was manifested in the reduction of the tourism business area or its direct closure due to the downturn of rural tourism, the reduction of the number of households engaged in tourism, and the reduction of the opportunities to participate in folk culture performance activities. The remaining two major obstacle factors were the area of farmland returned to forests (C_12_) and the cleanliness of the travel mode (C_13_). Under the long-standing strict implementation of policies and measures such as returning farmland to forests and closing mountains for greening, rural arable land has basically been retired to the greatest possible extent to give priority to ecological benefits and fully respect farmers’ wishes; this has greatly enriched the ecological background resources of the countryside. Under the influence of COVID-19, the development of rural tourism declined, and farmers participating in tourism chose to change their livelihood type to cope with the impact. Some farmers chose to work in neighboring counties and cities, and the expansion of the scope of their livelihood activities inevitably increased the cost of travel and environmental pollution, which directly affected the ecological environment of rural territorial systems.

## Discussion

Rural tourism has become an important driving force for the promotion of the economic development of ethnic areas, and the COVID-19 pandemic has had a tremendous impact on the development of rural tourism and even on socio-economics around the world; thus, determining how to realize the stable development of tourism in the rural territorial systems is the core issue facing sustainable development^42,50^. Rural resilience is the basis for the smooth operation of the rural territorial systems, and its study can help to grasp the characteristics and laws of the evolution of the rural territorial systems under different perturbations, identify the obstacles restricting the development of the countryside at different times, and formulate a path for tourism village resilience enhancement according to the time and conditions. The ultimate goals are to stabilize the functions and operation of the rural territorial systems, and to promote the sustainable development and realize the revitalization of the countryside.

The findings of this research revealed that the influx of tourism factors has led to the restructuring of the structure and function of the rural territorial systems, with corresponding changes in the structure of employment, social relations, land use, etc. The overall results have been positive, with an increase in the resilience of the countryside. Tourism village resilience was found to be the strongest for tourism-led farmers and the weakest for agriculture-led farmers. With the development of rural tourism, the rural industrial structure has changed^55^. The countryside began to emerge hotels, small supermarkets, restaurants and other tourism service industries, and this type of production activities and traditional agricultural production activities are different, with higher income returns, but also requires more capital investment, some farmers abandoned the cycle of the long, low-benefit agricultural production activities, and then choose a short cycle, high-benefit tourism service activities^56^. In addition, in the process of tourism participation, farmers’ access to information is broadened, their behavioral cognition is enhanced, their ecological awareness is increased, and their behavioral activities are more conducive to the development of the rural territorial system^57^. Overall, tourism participation has a significant positive effect on tourism village resilience enhancement from the farmers’ perspective, which is consistent with the findings of previous studies^56,58^.

In contrast, the COVID-19 shock had a negative effect on the rural territorial systems.Following the outbreak of COVID-19, the Government took measures such as blockade and quarantine, which helped to protect people from the disease but affected their livelihoods^59^. This is when the rural system becomes less stable, tourism development declines, the tourist village becomes less resilient, and farmers choose to transform their livelihoods in response to perturbations^60^. In addition, the increased energy consumption of farmers at home and the weakened awareness of ecological and environmental protection have had an impact on the ecological environment of the rural territorial system.Our results were similar to those of previous studies that concluded that COVID-19 epidemic had a strong impact on the tourism industry and a negative effect on the resilience of the tourist countryside from the farmers’ perspective^61–63^.

### Strengths and limitations

The main contribution of this study is its emphasis on the unity between the concept of PLE and the essence of resilience theory. Furthermore, the inclusion of the concept of PLE in the analytical framework of the resilience of tourism villages expands the research scope. Moreover, targeted research from the perspective of farmers can expand the micro-perspective of tourism village resilience research, thus enriching the connotation of tourism villageresilience.

While rural territorial systems actors include both the community and farmers, this study did not analyze the resilience in tourism villages from the community perspective. In future research, it will be necessary to synthesize these two perspectives to explore whether there are correlations and differences between the scales.^[31]^ Moreover, due to spatial limitations, the evolution mechanism of ttourism village resilience in ethnic areas was not clarified, which requires further consideration in subsequent research.

## Conclusions and policy implications

### Conclusions

Under the positive and negative influences of rural tourism and COVID-19, the resilience of tourism villages in Xiangxi Prefecture from the perspective of farmers has undergone evolution characterized by a steady increase followed by a slight decrease. The change trends of resilience in all dimensions were basically consistent with the overall tourism village resilience evolution trend, and the overall resilience level fluctuated and increased. During the undeveloped tourism period, the elements in the rural territorial systems were in a traditional symbiotic and equilibrium relationship. The livelihood of farmers was primarily based on the traditional way of living. Thus, the resilience of tourism villages in Xiangxi Prefecture was at a low level overall. During the period of the normalization of rural tourism, under the influence of positive tourism effects, cultural and idle material resources were revitalized and utilized, and the overall tourism village resilience rose to a medium level. After the outbreak of the COVID-19 pandemic, the rural territorial systems suffered serious impacts, the participation of farmers in tourism declined, and the level of tourism village resilience decreased.Differences were found in the resilience of the tourism villages in Xiangxi Prefecture from the perspective of different types of farmers. Tourism-led farmers had the strongest resilience, while agriculture-led farmers had the weakest resilience. The obstacles to the evolution of the resilience of tourism villages in Xiangxi Prefecture from the perspective of farmers showed a convergence trend.

### Policy implications

To enhance the level of resilience of tourism villages, the following aspects should be considered: (1) Government macro-control and policy support. In the face of rural tourism or exogenous perturbations, the government should play a macro-control role in tourism villages according to the positive and negative effects of the perturbation. The timely formulation of relevant policies can amplify the positive effects and dissipate the negative effects.(2) Enhanced information propaganda and active guidance from the community. At the community level, tourism-related policies can be interpreted, the spirit of the policies can be conveyed to farmers through collective meetings, bulletin boards, radio broadcasts, etc., and farmers can be actively guided to participate in tourism.(3) Farmers taking the initiative to seize opportunities and adapt to shocks. Thus, farmers should actively take the initiative to reasonably utilize the advantages of the main body and participate in rural tourism. They should reasonably increase the number of families participating in tourism and expand the tourism business area, and should actively respond to policies related to returning farmland to forests.

## Data Availability

The associated dataset of the study is available upon request to the corresponding author.
